# MRI-based radiomics approach for the prediction of recurrence-free survival in triple-negative breast cancer after breast-conserving surgery or mastectomy

**DOI:** 10.1097/MD.0000000000035646

**Published:** 2023-10-20

**Authors:** Jingwei Zhao, Qi Zhang, Muqing Liu, Xinming Zhao

**Affiliations:** a Department of Diagnostic Radiology, National Cancer Center/National Clinical Research Center for Cancer/Cancer Hospital, Chinese Academy of Medical Sciences and Peking Union Medical College, Beijing, China; b Department of Radiology, Chaoyang Central Hospital, Chaoyang, China.

**Keywords:** radiomics, recurrence-free survival, triple-negative breast cancer

## Abstract

To explore the value of a radiomics signature and develop a nomogram combined with a radiomics signature and clinical factors for predicting recurrence-free survival in triple-negative breast cancer patients. We enrolled 151 patients from the cancer imaging archive who underwent preoperative contrast-enhanced magnetic resonance imaging. They were assigned to training, validation and external validation cohorts. Image features with coefficients not equal to zero in the 10-fold cross-validation were selected to generate a radiomics signature. Based on the optimal cutoff value of the radiomics signature determined by maximally selected log-rank statistics, patients were stratified into high- and low-risk groups in the training and validation cohorts. Kaplan–Meier survival analysis was performed for both groups. Kaplan–Meier survival distributions in these groups were compared using log-rank tests. Univariate and multivariate Cox regression analyses were used to construct clinical and combined models. Concordance index was used to assess the predictive performance of the 3 models. Calibration of the combined model was assessed using calibration curves. Four image features were selected to generate the radiomics signature. The Kaplan–Meier survival distributions of patients in the 2 groups were significantly different in the training (*P* < .001) and validation cohorts (*P* = .001). The C-indices of the radiomics model, clinical model, and combined model in the training and validation cohorts were 0.772, 0.700, 0.878, and 0.744, 0.574, 0.777, respectively. The C-indices of the radiomics model, clinical model, and combined model in the external validation cohort were 0.778, 0.733, 0.822, respectively. The calibration curves of the combined model showed good calibration. The radiomics signature can predict recurrence-free survival of patients with triple-negative breast cancer and improve the predictive performance of the clinical model.

## 1. Introduction

Worldwide, breast cancer (BC) ranks first among all types of cancers in women and is the main cause of death in women with cancer.^[1]^ Triple-negative breast cancer (TNBC) is a subtype of BC.^[2]^ Patients with TNBC tend to be prone to recurrence and have a poorer prognosis than those with other types of BC.^[3]^ It is worth noting that the prognosis of patients with TNBC who undergo breast-conserving surgery (BCS) is not worse than that of mastectomy or even better.^[4–6]^ Predicting the risk of recurrence in patients with TNBC after BCS or mastectomy has important clinical significance, which helps clinicians develop personalized treatments for patients with TNBC. TNM staging and pathological grade were associated with patient prognosis of patients.^[7]^ However, histopathological classification fails to provide sufficient predictive value.^[8]^ They are not sufficient for personalized treatment because breast cancer is heterogeneous and intratumoral heterogeneity is poorly understood.^[9]^ TNBC heterogeneity affects the prognosis of TNBC patients Therefore, accurately stratifying the recurrence risk in patients with TNBC is essential for the development of personalized treatments.^[10]^

Radiomics can quantitatively transform medical images into features that potentially reflect tumor heterogeneity. Many recent studies have demonstrated the potential of radiomics for clinical applications. A study has demonstrated that texture features extracted from MR images are valuable for identifying the pathological grade of hepatocellular carcinoma (HCC), with poorly differentiated HCC being more heterogeneous than well-differentiated HCC.^[11]^ Radiomics can also be used to predict HCC prognosis. Features extracted from MR images are associated with the early recurrence of liver cancer, which can help clinicians develop personalized treatments.^[12]^ Radiomics also provides clinical value in breast cancer. Owing to the high soft-tissue resolution of magnetic resonance imaging (MRI), its sensitivity and specificity for the detection and diagnosis of breast cancer is the highest among all modalities.^[13]^ Based on the features extracted from MRI images, Dong et al^[14]^ built a model using logistic regression to predict axillary lymph node metastasis with an accuracy of 85 %. Many studies have demonstrated that radiomics can predict the prognosis of malignant tumors such as gastric cancer,^[15]^non-small-cell lung cancer,^[16,17]^ and clear cell renal cell carcinoma.^[18,19]^

This study aimed to establish a combined model based on image features extracted from preoperative MR images to predict recurrence-free survival (RFS) in patients with TNBC.

## 2. Methods

### 2.1. Patients selection

**Figure 1.**
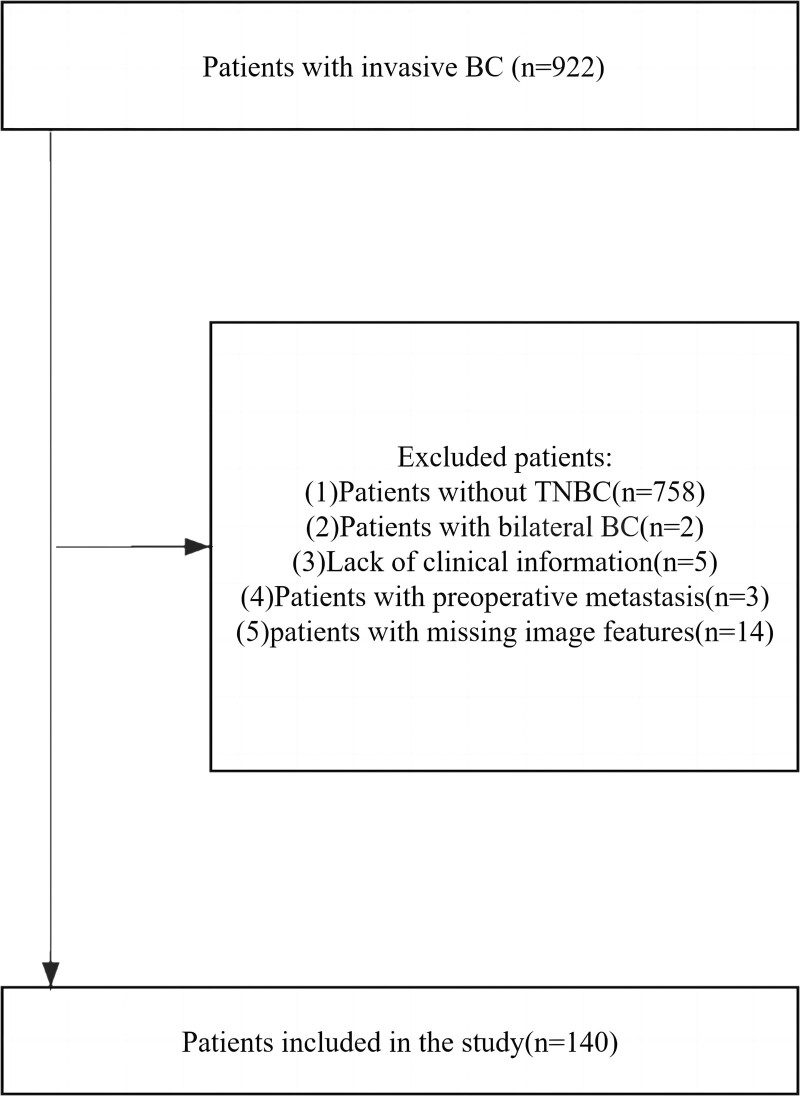
The flow chart for patient selection.

The data were acquired from the cancer imaging archive (TCIA).^[20]^ TCIA is a publicly available database that has been used in many previous studies.^[21–23]^ The collection^[24]^ we used contains clinical data and imaging features of 922 patients with invasive BC who underwent preoperative contrast-enhanced magnetic resonance imaging at Duke Hospital from 1 January 2000 to 23 March 2014.^[25]^ The clinical data of the patients included in this study included age, T stage, N stage, pathological grade, follow-up period, and recurrence during follow-up. RFS was defined as the period from the date of surgery to the date of recurrence or last follow-up. Patients were included in this study based on the following criteria: Pathologically confirmed TNBC; Unilateral BC; Patients who underwent BCS or mastectomy. The exclusion criteria were as follows: Lack of clinical information; Patients with preoperative metastasis; Missing image features. As all data involved in this study were anonymous and publicly available, the approval of the institutional review board was exempt, as in previous studies.^[21–23]^ The process of patient selection is outlined by the flow chart as shown in Figure 1. Based on these criteria, 140 patients were enrolled in this study.

### 2.2. Features selection and construction of radiomics model, clinical model, and combined model

A total of 529 imaging features of these 140 patients were downloaded from TCIA. These features were extracted from the pre-contrast, first post-contrast, and subtracted images (the first post-contrast images were subtracted from the pre-contrast images). The radiologist marked a bounding rectangle on each slice to identify the cuboid containing the lesions. Using the annotated cuboid, a fuzzy C-means automatic segmentation method, previously employed in studies for segmenting breast lesions^[1]^, was used to obtain the tumor mask. The lesion region was segmented using the subtracted images, confined within the boundaries of the annotated cuboid, as indicated by the radiologist. To generate the breast mask, the polynomial curve fitting algorithm was employed to remove the chest region, and the breast region was detected through a combination of global thresholding and an active contour method. Moreover, the fuzzy C-means method was utilized to automatically generate masks for the fibroglandular tissue (FGT) from both the T1-nonfat saturated images and the first post-contrast images. Consequently, each patient had 4 masks. Five Hundred Twenty-Nine image features were extracted based on these masks including 10 categories: Breast and FGT volumetric features(n = 5); Tumor size and morphology features(n = 10); FGT enhancement (n = 82); Tumor enhancement (n = 30); Combining tumor and FGT enhancement(n = 18); FGT enhancement texture (n = 176); Tumor enhancement texture (n = 135); Tumor enhancement spatial heterogeneity (n = 4); FGT enhancement variation (n = 34); Tumor enhancement variation (n = 35). At a ratio of 7:3, the patients were randomly assigned to the training and validation cohorts. The patients in the training cohort were used to construct 3 models. To reduce redundancy, correlation analysis was first performed on these 529 image features, and those with correlation coefficients > 0.8 were excluded. The image features that were not excluded were high-throughput features that could lead to overfitting. To overcome this problem, least absolute shrinkage and selection operator (LASSO) COX regression modeling was applied to select features. The optimal lambda value was determined using 10-fold cross-validation, and image features whose coefficients were not equal to zero were selected for further analysis. The selected features were multiplied by their coefficients and the results were added to obtain the radiomics signature (Rad-score). Clinical factors (*P* < .05) in univariate and multivariate analyses were incorporated into the clinical model. COX regression was performed to introduce the Rad-score into the clinical model to construct a combined model. The nomogram of the combined model was plotted.

### 2.3. Model assessment

The chi-square test, Fisher exact test, or *t* test was used to compare clinical factors between the training and validation cohorts. In the training cohort, the optimal cutoff value of the radiomics model was calculated according to maximally selected log-rank statistics. The patients were divided into low- and high-risk groups based on this cutoff value. Kaplan–Meier survival analysis was performed for both groups. The Kaplan–Meier survival distributions of the 2 groups were compared using the log-rank test. The cutoff value determined in the training cohort was then applied to the validation cohort. Patients in the validation group were divided into 2 groups. Kaplan–Meier survival analysis and log-rank tests were performed, as in the training cohort. The concordance index (C-index) was used to evaluate the predictive performance of the 3 models in the 2 cohorts. The predictive performances of the 3 models were compared using the likelihood ratio test. Calibration curves were used to calibrate the combined model.

### 2.4. External validation

To externally validate our findings, another collection (https://doi.org/10.7937/K9/TCIA.2016.QHsyhJKy) of 64 BC patients from TCIA was employed. Based on the inclusion and exclusion criteria, 53 patients without TNBC were excluded. Elven patients were included in the external validation cohort. The patients in the external validation cohort were divided into low- and high-risk groups based on the cutoff value calculated in the training cohort. Kaplan–Meier survival analysis and log-rank tests were performed, as in the training cohort. The predictive performances of the 3 models were compared using the likelihood ratio test. Calibration curves were used to calibrate the combined model.

### 2.5. Statistical analysis

Statistical analyses were conducted using R software (version 4.0.3, https://www.r-project.org/). Chi-square or Fisher exact test was used for categorical variables. A *t* test was used to analyze continuous variables. The “caret” package was used to calculate correlation coefficients. The “survival” package was used to perform COX regression for univariate and multivariate analyses and to calculate and compare the C-index of the 3 models. The “survminer” package was used for the maximally selected log-rank statistics. The “glmnet” package was used for LASSO-cox regression on high-dimensional data. The “rms” package was used to plot the nomogram.

## 3. Results

### 3.1. Patient characteristics and survival outcomes

The 140 patients in our study had a median follow-up period of 48 months (5–116 months), and 23 of them experienced recurrence. Among them, 3 patients had local recurrence, 19 patients had distant metastasis, and 1 patient had both local recurrence and distant metastasis. The median follow-up time of the 23 patients with recurrence was 15 months (7 months–61 months). Ninety-eight and 42 patients were randomly assigned to the training and validation cohort. The clinical characteristics of the patients in the 2 cohorts are shown in Table 1. There were no significant differences in these clinical factors between patients in the 2 cohorts.

**Table 1:** Clinical factors in training and validation cohorts.

	Training cohort (n = 98)	Validation cohort (n = 42)	*P* value
Age	50.61+_11.23	51.02+_12.02	.847
T stage			.534
T_1_	40	16	
T_2_	45	21	
T_3_	10	2	
T_4_	3	3	
N stage			.600
N_0_	61	25	
N_1_	27	10	
N_2_	6	3	
N_3_	4	4	
Histologic grade			.800
G_1_	1	1	
G_2_	28	11	
G_3_	69	30	
Surgery type			.461
Breast-conserving surgery	47	23	
Mastectomy	51	19	

### 3.2 Construction of radiomics model, clinical model and combined model

**Figure 2.**
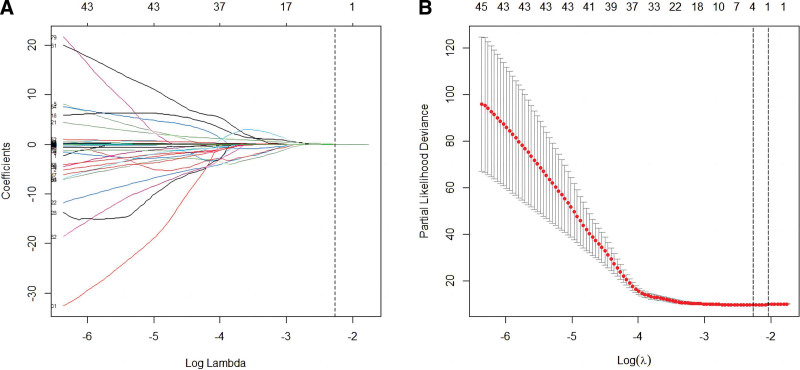
Features selection. (A) LASSO coefficient profiles of the 140 features. (B) Selecting the optimal lambda (Log (Lambda) = −2.267, Lambda = 0.10358) using 10-fold cross-validation. LASSO = least absolute shrinkage and selection operator.

After excluding features with correlation coefficients > 0.8, 153 features were included in the subsequent LASSO-COX regression. LASSO-Cox regression was performed on 153 features, and 4 features were screened out (Fig. 2).

**Figure 3.**
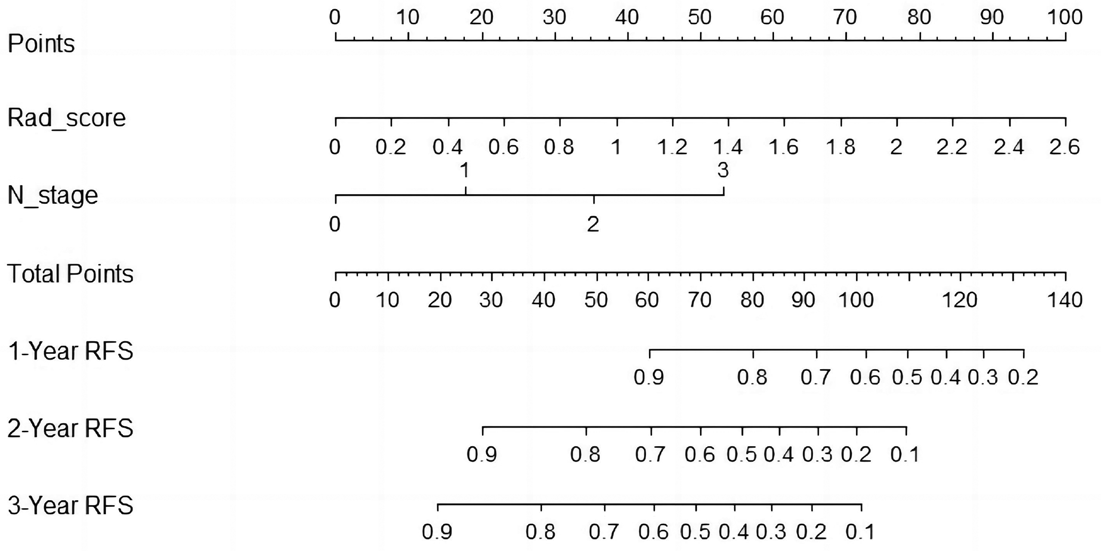
Nomogram constructed with N stage and Rad-score.

The formula of Rad-score:

Rad-score = 0.011737086*Median_Euler_No_Tumor,

+0.029262951*Uptake_rate_from_char_curv,

+0.000014*signal enhancement ratio (SER)_Total_tumor_vol_cu_mm,

+0.0000117*SER_map_kurtosis_tumor,

After univariate analysis of the clinical factors, The *P* values of the T and N stages were both < .05, as determined by univariate analysis. When these 2 features were further incorporated into the multivariate analysis, the *P* value of the N stage was still < .05, but the *P* value of the T stage was > .05. Therefore, N stage was used to construct the clinical model (Table 2). After multivariate analysis of N stage and Rad-score, N stage and Rad-score were identified as independent risk factors. Therefore, N stage and Rad_score were integrated into the combined model. The nomogram of the combined model was plotted (Fig. 3).

**Table 2:** Univariate and multivariate analysis of clinical factors.

Clinical factors	Univariate		Multivariate	
	Hazard ratio (95%CI)	*P* value	Hazard ratio (95%CI)	*P* value
Age	0.967 (0.884, 1.058)	.464		
T stage				
T_1_	Ref			
T_2_	1.070 (0.326, 3.507)	.911	2.283 (0.525, 9.935)	.271
T_3_	3.961 (1.061, 14.785)	.041	4.046 (0.957, 17.110)	.057
T_4_	3.860 (0.446, 33.400)	.220	12.274 (0.918, 164.054)	.058
N stage	Ref			
N_0_				
N_1_	1.014 (0.262, 3.927)	.984	0.631 (0.121, 3.296)	.585
N_2_	5.104 (1.310, 19.894)	.019	4.535 (0.791, 25.990)	.090
N_3_	14.058 (3.466, 57.018)	.0002	20.336 (3.499, 118.191)	.001
Histologic grade				
G_1_ or G_2_	Ref			
G_3_	1.311 (0.423, 4.065)	.639		
Surgery type				
Breast-conserving surgery	Ref			
Mastectomy	1.917 (0.695, 5.286)	.208		

### 3.3. Model assessment

**Figure 4.**
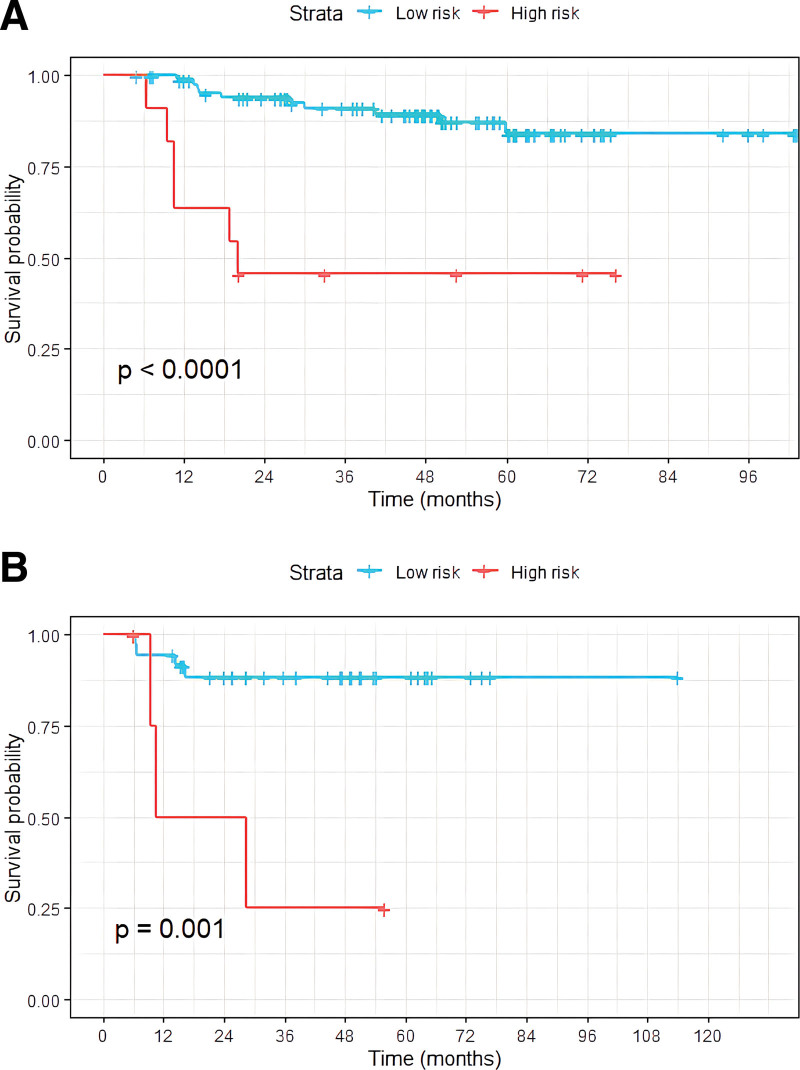
Kaplan–Meier survival analyses of two groups in the training cohort (A) and the validation cohort (B).

**Figure 5.**
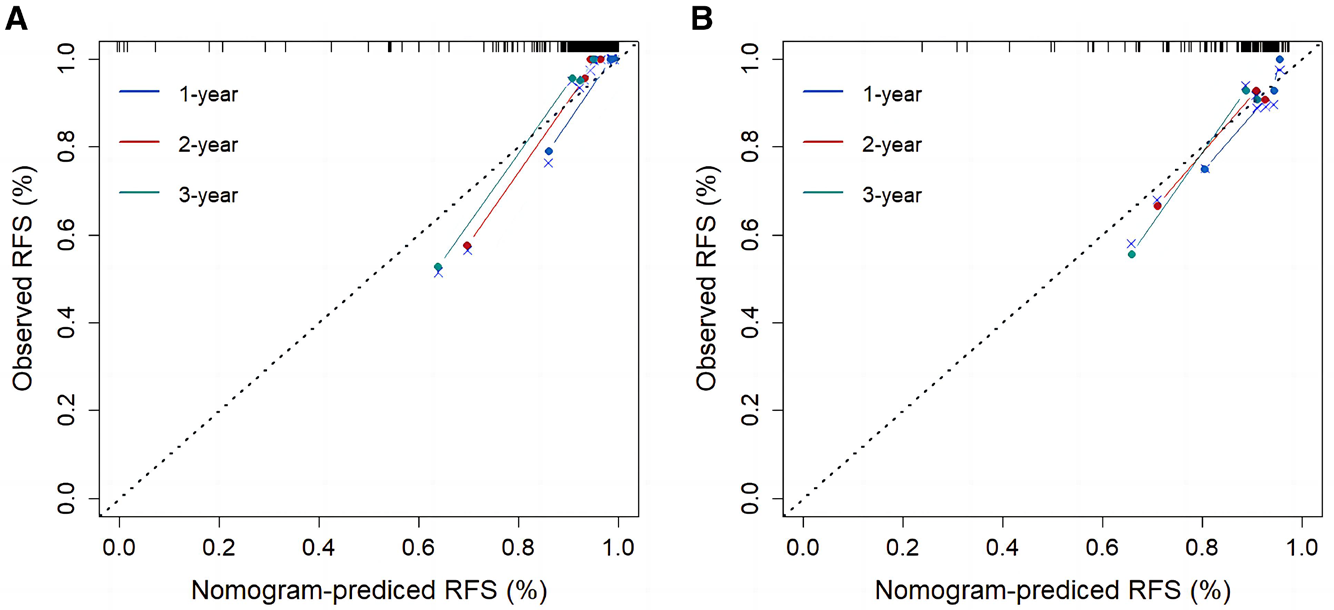
Calibration curves of the combined model for the (A) training and (B) validation cohorts.

According to the Rad-score cutoff value (0.61), patients with a Rad-score greater than the cutoff value were assigned to the high-risk group, and those with a Rad-score lower than the cutoff value were assigned to the low-risk group. There was a significant difference in the Kaplan–Meier survival distributions between the 2 groups (*P* < .001), as shown in Figure 4. According to the cutoff value (0.61), patients in the validation cohort were divided into low- and high-risk groups. The Kaplan–Meier survival distributions of the 2 groups also showed significant differences (*P* = .001), as shown in Figure 4. The C-indices of the 3 models are listed in Table 3. After adding the Rad-score, the C-index of the clinical model significantly improved in the training (from 0.700–0.878) and validation cohorts (from 0.574–0.777). The calibration curves of the combined model are shown in Figure 5. There was high consistency between the observed and predicted 1, 2, 3 years RFS.

**Table 3:** C-index of the three models.

	C-index (95% CI)	
	Training cohort (n = 105)	Validation cohort (n = 46)
Radiomics model	0.772 (0.648, 0.896)	0.744 (0.560, 0.928)
Clinical model	0.700 (0.563, 0.837)	0.574 (0.357, 0.790)
Combined model	0.878 (0.798, 0.986)	0.777 (0.627, 0.927)

CI = confidence interval, C-index = concordance index.

### 3.4. External validation

**Figure 6.**
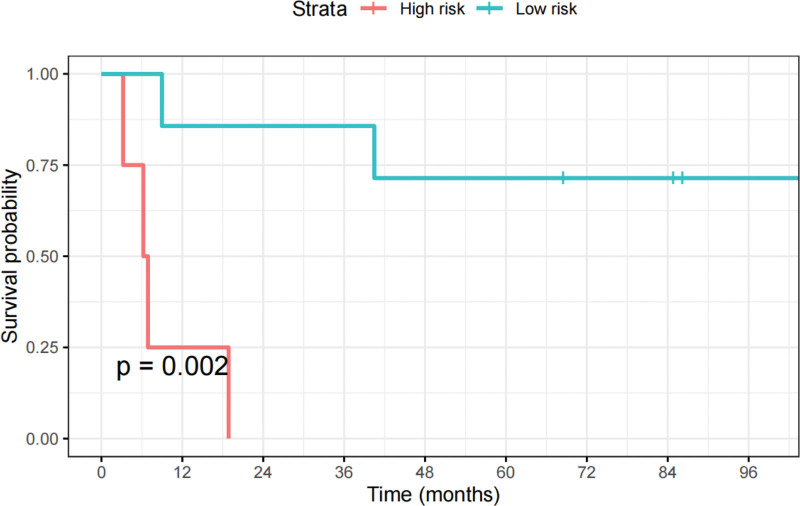
Kaplan–Meier survival analyses of two groups in the external validation cohort.

**Figure 7.**
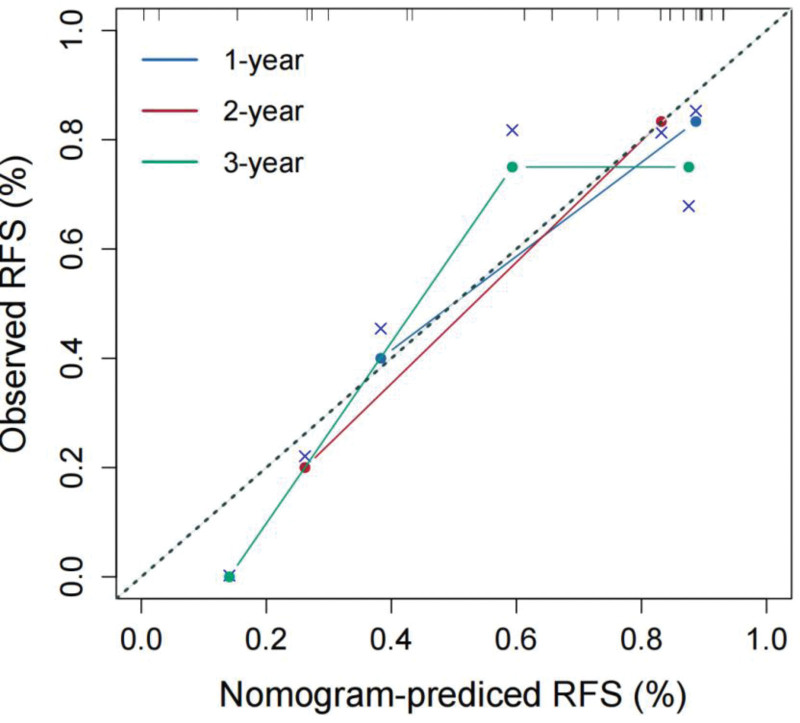
Calibration curves of the combined model for the external validation cohort.

According to the cutoff value (0.61), patients in the external validation cohort were divided into low- and high-risk groups. The Kaplan–Meier survival distributions of the 2 groups also showed significant differences (*P* = .002), as shown in Figure 6. The C-indices of the radiomics model, clinical model, and combined model in the external validation cohort were 0.778, 0.733, 0.822, respectively. After adding the Rad-score, the C-index of the clinical model significantly improved in the external validation cohort (from 0.733–0.822). The calibration curves of the combined model are shown in Figure 7. There was high consistency between the observed and predicted 1, 2, 3 years RFS.

## 4. Discussion

To predict the RFS of TNBC patients, we selected 4 image features extracted from preoperative MR images to construct a radiomics model. According to the recurrence risk, the optimal cutoff value of the Rad-score to stratify the patients into 2 groups was calculated. Patients in these 2 groups showed a significant difference in Kaplan–Meier survival distributions. N stage was selected to construct the clinical model after univariate and multivariate analyses. The combined model based on the Rad-score and N stage showed significantly higher predictive performance than the clinical model, indicating the added value of the Rad-score in the prediction of RFS.

In traditional practice, radiologists visually interpret medical images. The accuracy of interpretation is greatly affected by the experience of radiologists. Radiomics can be used to convert medical images into high-throughput quantitative valuable features. After analyzing these features, they can be combined with other patient information to improve predictive performance. Radiomics is expected to become a powerful tool for facilitating clinical decision-making.^[26]^ Several studies on breast cancer have confirmed the advantages of radiomics in differential diagnosis and prognosis prediction. Bickelhaupt et al^[27]^. used the LASSO algorithm to select features from MR images to differentiate malignant lesions from suspicious breast lesions. The area under the receiver operating curve (AUC) of the model constructed based on the selected features was 0.84, which was satisfactory. Fan et al^[28]^ constructed a model to predict molecular subtypes in a study that investigated the relationship between DCE-MRI features and molecular subtypes. The predictive performance of the model was evaluated using AUC. The AUC of distinguishing each subtype exceeded 0.8.

In this study, we identified 4 features for the construction of a radiomics model. Among the 4 selected features, SER_map_kurtosis_tumor is extracted from the signal enhancement ratio map. It describes the shape of the gray-level distribution in the delineated tumor area of the SER map. A higher value of SER_map_kurtosis_tumor indicates that the distribution of the gray-level is more concentrated and more close to the mean value. Median_Euler_No_Tumor describes the median values of Euler numbers computed for each slice. These 2 features may reflect the heterogeneity of TNBC. A previous study showed that SER_map_kurtosis_tumor is related to BC prognosis, which is consistent with the results of our study.^[29]^ SER_Total_tumor_vol_cu_mm describes the total volume of enhancing voxels above the initial enhancement level of 70%. One group found it beneficial to distinguish between malignant and benign breast lesions. They demonstrated that malignant tumors had a higher SER_Total_tumor_vol_cu_mm. This may reflect the malignancy of the breast lesions, which is related to the prognosis of TNBC.^[30]^ Uptake_rate_from_char_curv describes the rate at which the lesions reach peak enhancement. It has been shown to distinguish malignant from benign breast lesions.^[31]^ We also found that Uptake_rate_from_char_curv was associated with the prognosis of patients with TNBC. Patients were successfully assigned to 2 groups based on the optimal cutoff value of the Rad-score. The Kaplan–Meier survival distributions of patients in these 2 groups were significantly different in both the training and validation cohorts. The C-index of the radiomics model was 0.772 and 0.744 in the training and validation cohorts, respectively, indicating that the Rad-score has prognostic value for patients with TNBC.

Among the clinical factors, N stage was introduced into the clinical model as a risk factor after univariate and multivariate analyses. The lymph node status of BC patients is a risk factor for poor prognosis. Lymph node metastasis is associated with poor prognosis in patients with TNBC. Yang et al^[32]^ found that N stage is related to the RFS of patients with BC, which is consistent with our study. After the multivariate analysis, the Rad-score and N stage were integrated to construct a combined model. The predictive performance of the combined model was improved compared with that of the clinical model in the training (from 0.700–0.878), validation (from 0.574–0.777) and external validation (from 0.733–0.822) cohorts. This result suggests that the radiomics model has complementary value to clinical models for predicting RFS of patients with TNBC. In previous studies, predictive performance was improved when the radiomics model was combined with other models, which is similar to our results.^[33,34]^ The calibration curves of the combined model showed good calibration.

Our study had certain limitations. First, the number of patients involved in our study was relatively small. Therefore, it would be beneficial to conduct a larger cohort study to further validate our findings. Because our study was retrospective, bias is inevitable. Further prospective studies are required to confirm our findings.

## 5. Conclusion

The radiomics model based on image features from preoperative MR images is a prognostic factor for patients with TNBC and can predict the RFS of patients with TNBC. The combined model based on the Rad-score and N stage showed a significant improvement in predictive performance compared with the clinical model. Our research demonstrates that radiomics complements the accuracy of predicting prognosis in patients with TNBC, providing a potential decision-making tool for clinicians.

## Author contributions

**Conceptualization:** Qi Zhang, Xinming Zhao.

**Data curation:** Jingwei Zhao, Muqing Liu.

**Formal analysis:** Jingwei Zhao.

**Funding acquisition:** Xinming Zhao.

**Investigation:** Jingwei Zhao.

**Methodology:** Jingwei Zhao.

**Project administration:** Qi Zhang.

**Resources:** Jingwei Zhao.

**Software:** Jingwei Zhao.

**Supervision:** Xinming Zhao.

**Validation:** Jingwei Zhao, Muqing Liu.

**Visualization:** Jingwei Zhao.

**Writing – original draft:** Jingwei Zhao.

**Writing – review & editing:** Qi Zhang, Xinming Zhao.
